# Rétraction: plagiat aggravé dans l'article *Aspergillose broncho-pulmonaire allergique lors d'un asthme réfractaire: à propos d'un cas clinique. Khalid Lahmadi, Hind El Youssi, Jawad Rochdi, BoughrineNawal, Er-Rami Mohammed. The Pan African Medical Journal. 2015;20:350. (doi:10.11604/pamj.2015.20.350.6572)*

**DOI:** 10.11604/pamj.2015.21.25.7022

**Published:** 2015-05-12

**Authors:** PanAfrican Medical Journal

**Affiliations:** 1 The Pan African Medical Journal, Yaoundé, Cameroun

**Keywords:** Plagiat, retraction, PAMJ, plagiarism, retraction, PAMJ

## Abstract

Cet article retracte l'article "*Aspergillose broncho-pulmonaire allergique lors d'un asthme réfractaire: à propos d'un cas clinique. Khalid Lahmadi, Hind El Youssi, Jawad Rochdi, BoughrineNawal, Er-Rami Mohammed. The Pan African Medical Journal. 2015;20:350. (doi:10.11604/pamj.2015.20.350.6572)*"

## Rétraction

Nous tenons à notifier aux lecteurs de PAMJ, la rétraction du manuscrit Aspergillose broncho-pulmonaire allergique lors d'un asthme réfractaire: à propos d'un cas clinique (doi:0.11604/pamj.2015.20.350.6572) par Khalid Lahmadi, Hind El Youss, Jawad Rochdi, Boughrine Nawal et Er-Rami Mohammed du Laboratoire de Parasitologie, Hôpital Militaire Moulay Ismail de Meknès, Meknès, Maroc [1]. Au terme de notre investigation, il apparait de manière indiscutable que le manuscrit soumis par Khalid Lahmadi et al. et publié dans PAMJ le 10 Avril 2015, a été purement et simplement plagié par les auteurs. En effet, le manuscrit soumis par Khalid Lahmadi, Hind El Youss, Jawad Rochdi, Boughrine Nawal et Er-Rami Mohammed du Laboratoire de Parasitologie, Hôpital Militaire Moulay Ismail de Meknès, Meknès, Maroc et publié dans PAMJ est une copie conforme du manuscrit Aspergillose broncho-pulmonaire allergique: un diagnostic à évoquer lors d'un asthme réfractaire 24(2)- e82; doi:10.1016/j.mycmed.2014.01.089) par I. Tlamçani, S. Benjelloun, H. Deham, et M. Er-Rami publiée dans le Journal de Mycologie Médicale [2]. Par conséquent, nous retirons cet article de la littérature médicale en conformité avec les lignes directrices et les meilleures pratiques éditoriales du "Committee on Publication Ethics". Nous nous excusons auprès de notre public pour cette situation malheureuse.
